# Interfacial Deposition of Titanium Dioxide at the Polarized Liquid–Liquid Interface

**DOI:** 10.3390/ma15062196

**Published:** 2022-03-16

**Authors:** Karolina Kowalewska, Karolina Sipa, Barbara Burnat, Sławomira Skrzypek, Lukasz Poltorak

**Affiliations:** Electroanalysis and Electrochemistry Group, Department of Inorganic and Analytical Chemistry, Faculty of Chemistry, University of Lodz, Tamka 12, 91-403 Lodz, Poland; karolina.kowalewska@chemia.uni.lodz.pl (K.K.); karolina.sipa@chemia.uni.lodz.pl (K.S.); barbara.burnat@chemia.uni.lodz.pl (B.B.); slawomira.skrzypek@chemia.uni.lodz.pl (S.S.)

**Keywords:** ITIES, interfacial polycondensation, electrochemistry, voltammetry, interfacial modification, titanium dioxide synthesis

## Abstract

The interfacial polycondensation of titanium dioxide was studied at the bare and fiberglass membrane supported polarized liquid–liquid interface (LLI). Titanium dioxide synthesis was derived from the titanium (IV) tetrabutoxide (initially dissolved in the 1,2-dichloroethane) interfacial hydrolysis followed by its condensation. Experimental parameters, such as the pH of the aqueous phase and the influence of titanium alkoxide concentration in the organic phase on the electrochemical signal and material morphology, were investigated. The latter was achieved with fiberglass membranes used as the LLI support during TiO_2_ interfacial deposition. Cyclic voltammetry was used for the in situ studies, whereas scanning electron microscopy, energy-dispersive X-ray spectroscopy, and infrared spectroscopy were used during ex situ examination. The interfacial polycondensation reaction could be studied using electrified LLI and resulted in the material being a TiO_2_ film alone or film decorated with particles.

## 1. Introduction

Titanium dioxide (TiO_2_) is a material with a still-expanding list of applications. It can be used, e.g., to purify water and air, as a food additive, in coatings fabrication for self-cleaning surfaces, or as a UV filter in cosmetic products [1,2,3]. In this respect, TiO_2_ nanomaterials have become very popular due to relatively low price, non-toxicity, corrosion resistance, or well-established protocols to tune their surface physico-chemistry (e.g., porosity) [4]. TiO_2_ synthesis can be derived from many protocols such as the sol–gel processing, hydrothermal method, solvothermal method, and physical and chemical vapor deposition [5]. TiO_2_ thin films processing, with significant potential to be applied in electronic and magneto-electric devices, is challenging. Their formation at the planner liquid–liquid interface (LLI) is an intuitive choice as a free-stating thin film may be obtained. This work aims at providing first insights into electrochemically formed TiO_2_ at the LLI.

Electrochemistry at the polarized LLI, alternatively called interface between two immiscible electrolyte solutions (ITIES), finds applications in material science, pharmacochemistry, physicochemistry, and analytical chemistry. ITIES allows the study of the interfacial charge transfer reaction in the form of ions or electrons crossing the soft junction. Polarized LLIs offer properties that are difficult or even impossible to be obtained with conventional electrochemical systems, including (i) the possibility to separate reactive chemical compounds between two immiscible phases, (ii) defect and crack free electrified interface deprived of preferential nucleation sites, or (iii) asymmetric and amphiphilic properties that may be harvested during interfacial deposition reactions. Moreover, electrified LLIs (alone or in the presence of interfacial deposits) can be investigated with all available electrochemical techniques [6].

The placement of TiO_2_-based objects at the ITIES is limited to a few examples. In two elegant works, Jensen et al. [7] and later Plana and Fermin [8] showed that preformed TiO_2_ nanoparticles self-assembled at the electrified water—1,2-dichloroethane (1,2-DCE) interface could be used to generate photocurrent responses when illuminated with laser light. Another report describes coumarin 343 dye TiO_2_ nanoparticles complex interfacial adsorption studied with surface second harmonic generation technique [9]. The intuitive combination between conventional LLI and TiO_2_ nanoparticles are Pickering emulsions that can be frequently found in everyday products (e.g., stabilized sun creams) or the scientific literature [10,11,12]. The sol–gel processing of the TiO_2_ can occur at the LLI. Titanium alkoxides are usually dissolved in organic solvent immiscible with water providing self-dissociation reactions products (H_3_O^+^ or OH^−^) catalyzing hydrolysis and condensation reactions happening at the LLI [13,14,15]. To the best of our knowledge, such reactions have not been investigated at the electrified LLI to date.

This work aims to study TiO_2_ interfacial polycondensation at polarized LLI. This project is derived from our experience related to in situ ITIES modification with silica [16] and polyamide-based materials [17]. In this respect, we separated TiO_2_ precursor (titanium (IV) butoxide dissolved in the organic phase) from the aqueous phase, which is a solution of a background electrolyte at fixed pH. The following parameters were investigated: pH of the water phase, the effect of titanium (IV) butoxide concentration, and the effect of scan rate on the electrochemical signal. Synthesized material was removed from the interface and characterized using infrared spectroscopy. Finally, we used the fiberglass membranes as the LLI support during TiO_2_ synthesis. These were then studied using SEM and EDX. This communication reports on the possibility to form TiO_2_ derived from sol–gel processing at the eLLI. After the Materials and Methods section, which lists all chemicals and provides a brief description of the employed methodology, we discussed the possible mechanism governing the electrochemically assisted TiO_2_ interfacial polycondensation. We then correlated our understanding of the investigated platform with electrochemical data obtained using ion transfer voltammetry. Finally, we designed the experiment that allowed for the interfacial deposition of TiO_2_ over porous support placed within the LLI that was further subjected to comprehensive characterization.

## 2. Materials and Methods

### 2.1. Chemicals

Titanium (IV) butoxide (Ti[O(CH_2_)_3_CH_3_]_4_, reagent grade 97%, Aldrich Chemistry, Germany), sodium chloride (NaCl, for analysis, ChemPur, Poland), 1,2-dichloroethane (1,2-DCE, for analysis, POCH, Poland), 35–38% hydrochloric acid (HCl, for analysis, ChemPur, Poland), sodium hydroxide (NaOH, for analysis, ChemPur, Poland), phosphate buffer made according to appropriate weights of sodium hydrogen phosphate dihydrate (NaH_2_PO_4_ × 2H_2_O, for analysis, ChemPur, Poland), and sodium chloride (NaCl, for analysis, ChemPur, Poland) were used as received. The organic phase electrolyte BTPPA^+^TPBCl^−^ (bis(triphenylphosphoranylidiene)ammonium tetrakis(4-chlorophenylborate)) was synthesized using BTPPA^+^Cl^−^ (bis(triphenylphosphoranylidiene)ammonium chloride, 97% Sigma-Aldrich, Germany) and KTPB^+^Cl^−^ (potassium tetrakis(4-chlorophenyl), 98% Sigma-Aldrich, Germany) salts according to the recipe published elsewhere [18]. The aqueous phase was prepared from the demineralized water (Hydrolab system, Poland).

### 2.2. Methods

Electrochemical experiments: The TiO_2_ synthesis at the ITIES was studied in a dedicated macroscopic voltammetric glass cell (interface radii equal to 0.7 cm) (custom made by glass blower) equipped with a set of four electrodes consisting of two Ag/AgCl reference electrodes (Ag, 99.99%, Alfa Aesar, Germany) and two Pt counter electrodes (Pt, 99.9%, Sigma Aldrich, Germany). The potential interfacial difference was measured between Ag/AgCl wire immersed into the aqueous phase Luggin capillary filled with the aqueous phase with a fixed concentration of Cl^−^ ions and Ag/AgCl wire present inside the organic phase Luggin capillary filled with a solution of 10 mM NaCl and 10 mM BTPPA^+^Cl^−^ remaining in direct contact with the 1,2-DCE solution. All experiments were conducted using EmStat3+ equipped with a differential pulse amplifier from PalmSens (The Netherlands). Ion transfer voltammetry (potentiostatic cyclic voltammetry) was used to study the changes in the physicochemical properties of the polarized LLI at different TBOT organic phase % concentrations and different aqueous phase pH. The employed technique allowed for direct insights into ion transfer reactions happening at different polarization directions and interfacial electrical capacitance. The experimental scan rate value was 20 mV s^−1^ unless otherwise stated. Interfacial capacitance was extracted from the ion transfer voltammograms recorded at different potential scan rate values (5; 10; 15; 20; 25; 30 and 35 mV s^−1^). During all experiments, the LLI was always polarized from less positive to more positive potential values during the forward scan.

Infrared spectroscopy: TiO_2_ synthesized at the non-polarized LLI was collected and dried at room temperature. Dry powder was analyzed using the KBr pellet method with a Nexus FT-IR by Thermo Nicolet spectrometer (USA).

Scanning Electron Microscopy: The morphology of the unmodified and titanium dioxide modified glass fiber membranes was examined using Phenom G2 Pure scanning electron microscope (Phenom-World BV, Eindhoven, the Netherlands) operating with an accelerating voltage of 5 kV. The surface elemental composition analysis was performed using FEI Nova NanoSEM 450 microscope (Hillsboro, OR, USA) equipped with an EDS analyzer. Elemental analysis was carried out using an accelerating voltage of 10 kV.

## 3. Results and Discussion

### 3.1. TiO_2_ Formation at the Electrified Liquid–Liquid Interface—Consideration

**Figure 1:**
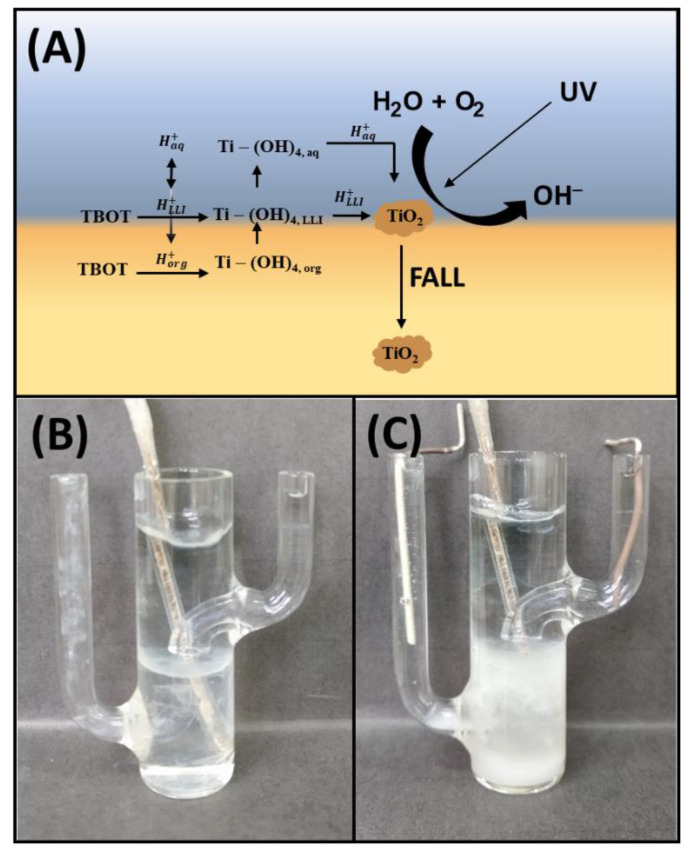
(**A**) Anticipated mechanisms laying behind the interfacial, electrochemically assisted TiO_2_ polycondensation. The photo of the electrochemical cell was taken for 0.5% TBOT (**B**) before and (**C**) after LLI polarization.

The composition of the immiscible phases used for the interfacial deposition of the TiO_2_ at the eLLI is shown in Figure 1A. The aqueous phase was the Britton–Robinson buffer providing H^+^ or OH^−^ needed for catalyzing tetrabutoxy titanium (TBOT) hydrolysis and condensation reactions together with other inorganic ions assuring high electric conductivity. The organic phase was the 1,2-DCE solution of the background, hydrophobic electrolyte (BTPPA^+^TPBCl^−^), and TBOT added at different % concentrations ranging from 0.01% to 1%. The interfacial polycondensation reactions occurring at the LLIs provide several routes that may lead to the formation of free-standing materials/films, out-printing the molecular properties of the hydrophobic and hydrophilic phases. As reported in a few elegant works [19,20], TiO_2_ can be formed at the LLI followed by sol–gel processing. Substrates, products, and catalysts of the hydrolysis and condensation reaction may undergo partitioning to the contacting phases, and whenever charged, this process is affected by the potential interfacial drops, which may be studied with all techniques offered by the electroanalytical toolbox. Figure 1B and Equations (1)–(11) depicts possible and simplified (100% efficient TBOT hydrolysis is assumed) reactions that may occur spontaneously and/or can be aided electrochemically at the LLI during TBOT hydrolysis and condensation [21,22]. These reactions are simplified to fully hydrolyzed TBOT and are defined based on our understanding of the interfacial region.

(1) $$Ti-{\left(O-R\right)}_{4,\;org}+4H_{2}O\to Ti-{\left(OH\right)}_{4,org}+4R-OH$$

(2) $$Ti-{\left(O-R\right)}_{4,\;LLI}+4H_{2}O\to Ti-{\left(OH\right)}_{4,LLI}+4R-OH$$

(3) $$Ti-{\left(OH\right)}_{4,org}\;\to Ti-{\left(OH\right)}_{4,LLI}$$

(4) $$Ti-{\left(OH\right)}_{4,LLI}\;\to Ti-{\left(OH\right)}_{4,aq}$$

(5) $$H_{aq}^{+}\;\to H_{org}^{+}$$

(6) $$OH_{aq}^{-}\;\to OH_{org}^{-}$$

(7) $$Ti-{\left(OH\right)}_{4,\;LLI}+Ti-{\left(OH\right)}_{4,\;LLI}\to {\left(OH\right)}_{3}-Ti-O-Ti-{\left(OH\right)}_{3,LLI}+H_{2}O$$

(8) $$Ti-{\left(OH\right)}_{4,\;LLI}+Ti-{\left(OH\right)}_{4,\;aq}\to {\left(OH\right)}_{3}-Ti-O-Ti-{\left(OH\right)}_{3,LLI}+H_{2}O$$

(9) $$Ti-{\left(OH\right)}_{4,\;LLI}+Ti-{\left(OH\right)}_{4,\;org}\to {\left(OH\right)}_{3}-Ti-O-Ti-{\left(OH\right)}_{3,LLI}+H_{2}O$$

(10) $$Ti-{\left(OH\right)}_{4,\;aq}+Ti-{\left(OH\right)}_{4,\;aq}\to {\left(OH\right)}_{3}-Ti-O-Ti-{\left(OH\right)}_{3,aq}+H_{2}O$$

(11) $${\left(OH\right)}_{3}-Ti-O-Ti-{\left(OH\right)}_{3,aq}\overset{precipitation}{\to }{\left(OH\right)}_{3}-Ti-O-Ti-{\left(OH\right)}_{3,LLI}\;$$

Hydrophobic TBOT soluble in most organic solvents was initially present in the organic phase where it can undergo hydrolysis (Equation (1)) triggered by the H^+^ or OH^−^ electrochemically transferred from the aqueous to the organic phase (Equations (5) and (6), respectively). Interfacial transfer of ions across the LLI can happen only when the appropriate interfacial potential difference is applied to the interfacial region. The presence of water molecules within the interfacial region is governed not only by the mutual solubility of the employed solvents (reported 1,2-DCE solubility in H_2_O is 0.085 mol·dm^−3^ whereas water in 1,2-DCE is 0.11 mol·dm^−3^ [23]) but also can be delivered to the organic phase as the cargo of inorganic ions which are not entirely deprived of the solvation shells when going from the aqueous to the organic phase [20]. This is especially valid for small cationic and anionic species such as Na^+^, Li^+^, K^+^, Cl^−^, Br^−^, etc. [24,25,26,27,28]. TBOT hydrolysis is also expected to happen within a region defined by the LLI thickness (Equation (2)). For both cases (Equations (1) and (2)), resulting titanium hydroxide species with high hydrophilicity are expected to partition from the organic phase to the interfacial region (Equation (3)) and further to the aqueous phase (Equation (4)) where their subsequent condensation reactions happen (Equation (7)—condensation within the plane of LLI; Equation (8)—condensation between the species from the LLI and the aqueous phase; Equation (9)—condensation between the species from the LLI and the organic phase; Equation (10)—condensation reactions happening in the aqueous phase). Butanol, which is the side product of the hydrolysis reaction, miscible with H_2_O and 1,2-DCE increases the mutual solubility of the concerned solvents, which in turn affects the thickness of the LLI and increases the availability of the substrates of the reaction. The TiO_2_ photocatalytic water reduction (caused by the daylight irradiation) leading to the formation of OH^−^ anions were also expected to contribute to the condensation reactions kinetics. The consequence of the electrochemically controlled ion transfer reaction happening in parallel and affecting the TBOT hydrolysis and condensation reactions initially lead to the interfacial formation of TiO_2_ (Figure 1B), which further precipitates into the organic phase (see Figure 1C).

### 3.2. Voltammetric Insights into Interfacial TiO_2_ Formation

**Figure 2:**
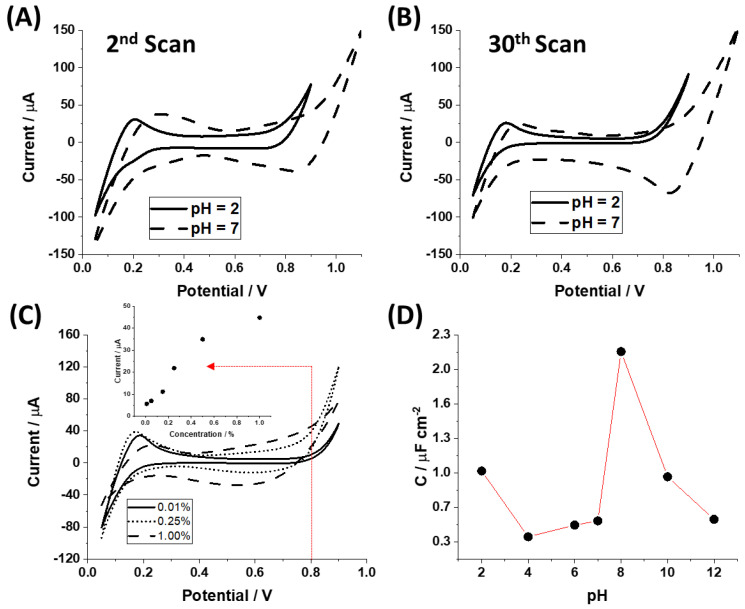
Ion transfer voltammograms (ITVs) recorded for 0.5% TBOT initially dissolved in the organic phase at pH of the aqueous phase equal to 2 (solid line) and 7 (dashed line) for the 2nd (**A**) and 30th (**B**) scan. (**C**) ITVs recorded for TBOT with the organic phase % concentration equal to 0.01% (solid line), 0.25% (dotted line), and 1.00% (dashed line). The second scan is shown. The inset in section (**C**) corresponds to positive ionic current falling for 0.8 V (indicated with the red, horizontal dashed line) recorded for different organic phase TBOT concentrations. pH of the aqueous phase was equal to 2. (**D**) The specific LLI capacitance derived from the voltammetric data plotted in function of the aqueous phase pH.

**Figure 3:**
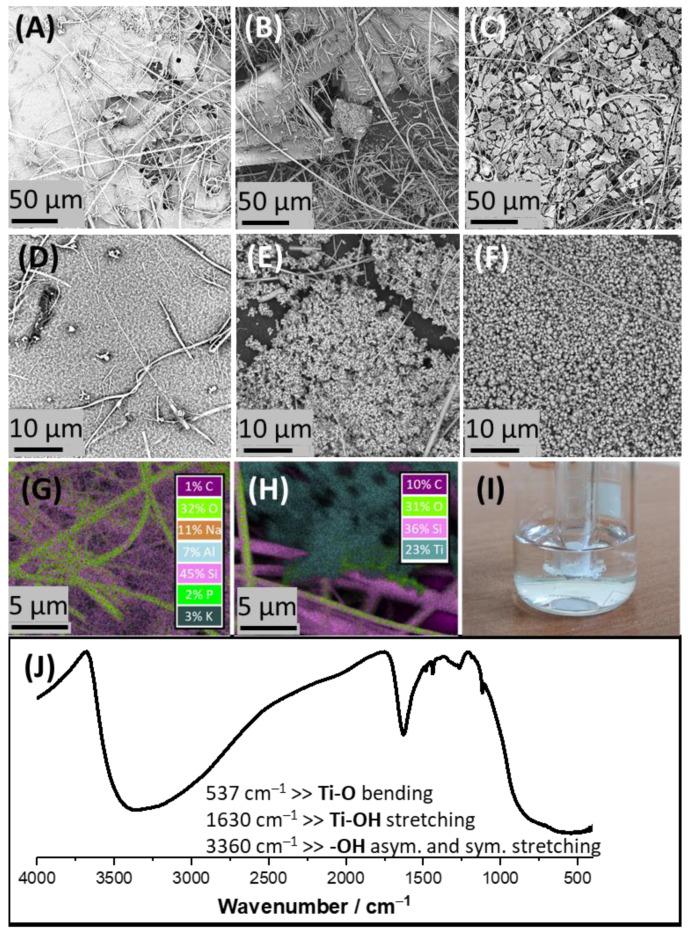
(**A**–**F**) The SEM micrographs recorded for the TiO_2_ collected from the LLI with 0.10%, 0.50%, and 1.00% of TBOT initially present in the organic phase; the pH of the aqueous phase was set to 8. (**G**,**H**) The EDS mapping micrographics recorded for the glass fiber membrane before and after modification (1.00% of TBOT in the organic phase) with TiO_2_, respectively. (**I**) The photo of a glass fiber membrane fixed to a glass tube. (**J**) The infrared spectrum recorded for the material collected from the LLI (main signals are attributed to the indicted absorption maxima wavenumbers).

The interfacial polycondensation of TiO_2_ at the polarized LLI was studied using ion transfer voltammetry. We investigated the effect of the electrochemical processing (the effect of the applied Galvani potential difference, voltammetric cycling, applied sweeping potential scan rate) and the chemical conditions (TBOT % concentration, pH of the aqueous phase) on the experimental output. Figure 2A,B shows the ion transfer voltammograms recorded for the 2nd and 30th cycle, respectively, recorded for two different pH values (2 and 7), assuring high and low concentrations of protons that may be transferred to the organic phase upon interfacial polarization. We observed that subsequent cycling and increasing % concentration of the TBOT in the organic phase affected the separation of the (i) capacitive currents within the available potential window and limiting currents on the (ii) positive and (iii) negative voltammetric scan potential ends. For the latter, both the intensity and the potential at which the background electrolyte ions started crossing the LLI have changed. This observation is in line with the increasing amount of the interfacially formed TiO_2_. At low pH values (around 2), the positive end of the potential window is limited by the interfacial transfer of H^+^ from the aqueous to the organic (positive current) and from the organic to the aqueous (negative current) phase [28,29]. Protons present within the LLI and in the organic phase were expected to accelerate hydrolysis and condensation reactions. This can be visualized in Figure 3C together with its inset representing the change in the current intensity recorded at 0.8 V. The potential interfacial difference for which an increase in positive current on the more positive potential window end was equal to 5% change, the capacitive current was attributed to the potential difference at which H^+^ started transferring from the aqueous to the organic phase [28]. We found that this potential was progressively shifting toward more negative potential values and was equal to 0.76 V for 0.01% TBOT, 0.49 V for 0.25% TBOT, and 0.33 V for 1.00% TBOT. This observation can be attributed to a few interconnected aspects: (i) increasing concertation of TBOT in the organic phase most probably facilitated the transfer of proton to the organic phase (indicated by the negative shift of the limiting current potential); (ii) high TBOT concentration leads to an autocatalytic effect on hydrolysis/condensation reactions as the concertation of protons in the interfacial layer increases (increasing intensities of the limiting current on the positive end—see inset of Figure 2C); (iii) presence of the butanol as a side product of the hydrolysis reaction changes the miscibility of the water and 1,2-DCE, and hence lower free Gibbs energy (energy needed to deprive the transferring ions of the solvation shells) of the proton transfer from the aqueous to the organic phase. Moreover, for high TBOT concentration (1.00%) for the aqueous phase with pH = 2 we observed the cyclic voltammograms with inclined limiting currents (originating from the H^+^ transfer on the more positive potential window side and Cl^−^ anions transfer on the less positive potential window side). This observation is attributed to the formation of a compact TiO_2_ based film, making a physical barrier directly existing at the LLI, which increases the resistance of the transferring ions.

An interesting observation is provided in Figure 2D showing the interfacial specific capacitance (µF·cm^−2^) plotted in function of the aqueous phase pH. The LLI formed between the aqueous phase, which is the Britton–Robinson buffer solution holding a fixed pH value and 0.5% TBOT solution dissolved in 5 mM BTPPA^+^TPBCl^−^ in 1,2-DCE was polarized at different voltammetric scan rates ranging from 5 to 35 mV·s^−1^. From the obtained voltammograms, we chose the potential difference region where no Faradaic reactions were occurring (data not shown). Next, we plotted the half of the forward and reversed capacitive currents separation in the function of the applied scan rate giving a linear dependency. The slope of the obtained curve with a unit of Farad (F) was further divided by the geometrical area of the cell used to support LLI (ø = 1.25 cm; A = 1.23 cm^2^). Finally, we plotted the resulting specific capacitance in the function of pH, providing a very characteristic pattern shown in Figure 2D. The obtained dependency resembles the relative rate of the Si-(OR)_4_ condensation reactions obtaining maximum values for the acidic environments and pH values falling for the pH range from 8 to 10 [30]. The capacitance of the system should be growing as the amount of interfacially accumulated charge increases. During interfacial condensation of TiO_2_, this will mainly originate from the increasing amount of interfacially accumulated charge in the form of background electrolyte salts. Miscibility of both phases, and hence, the thickness of the LLI increases as the condensation proceeds, and butanol is formed as the condensation reaction side products (dielectric properties of the “thick” LLI allow the accumulation of a higher amount of charge). Moreover, the isoelectric point of TiO_2_ is reported to be in the pH range from 5 to 7 [31], meaning that beyond this range, it is positively and negatively charged, respectively. According to the result shown in Figure 2D, most charges was accumulated at the LLI at pH around 8–10 and for pH < 3, which also coincide with the high amount of the deposited TiO_2_ material (visual observation). As such, we concluded that the dependency shown in Figure 2D correlate with the titanium alkoxide sol–gel condensation kinetics.

### 3.3. Interfacially Formed TiO_2_ Characterization

Figure 3A–F show SEM micrographs that were taken for the fiberglass membranes used as the eLLI support and further modified with TiO_2_ derived from the interfacial polycondensation reaction. The image of the cell used during TiO_2_ formation is depicted in Figure 3I and shows a beaker filled with the aqueous phase (pH = 8—chosen based on the result provided in Figure 2D), in which we immersed a glass tube filled with the organic phase ended with a fiberglass membrane capillary. The potential drop across such formed interface was defined by the aqueous and the organic phase composition (around 0.4 V). After experimental processing (deposition time was fixed to 30 min; pH of the aqueous phase was 8; the TBOT % concentration in the organic phase was 0.10%; 0.50% or 1.00%), fiberglass membranes were collected and analyzed using SEM and EDS. Figure 3A,D shows the TiO_2_ deposit formed at 0.10% TBOT concentration predominantly existing in the form of a porous film. White fibers, which are visible on all images, are made out of glass and make the volume of the used support (see Figure 3G for the elemental composition). A further increase in TBOT % concentration to 0.50% and 1.00% (see Figure 3B,E and Figure 3C,F, respectively) provided deposits with a shape of a film decorated generously with spherical TiO_2_ particles with diameters ranging from few tens to a few hundred nm. The nature of the formed material was studied with EDS (see Figure 3H showing the mapping of the formed flake) and infrared spectroscopy (see Figure 3J), further confirming the presence of TiO_2_. For the latter, the presence of the Ti–O bending absorption signal with a center at 537 cm^−1^; Ti–OH stretching mode with absorption peak maximum falling for 1630 cm^−1^, and very brad absorption of -OH groups spanning from 2500 to 3700 cm^−1^ confirms the nature of synthesized material [32,33]. This work has delivered the very first proof of concept studies showing the possibility to form TiO_2_ at the eLLI. In the future, we plan to improve the proposed methodology further to be able to control TiO2 interfacial polycondensation reactions electrochemically.

## 4. Conclusions

In this work, we provided the preliminary data confirming the possibility to spontaneously deposit TiO_2_ at the polarized LLI. Voltammetry was used to follow the sol–gel processing, whereas the electrochemically controlled interfacial ion transfer reactions (especially the transfer of H^+^ from the aqueous to the organic phase) presumably affected the condensation of TBOT hydrolysis products (Ti(OR)_X_(OH)_y_; x + y = 4). The specific capacitance of the LLI calculated from the voltammetric response of a system displayed the highest values falling for the acidic region and the slightly basic pH of the aqueous phase. It is proposed that obtained interfacial capacitance vs. the aqueous pH dependency correlate with the titanium alkoxide condensation reactions kinetics. Finally, we used the fiberglass membranes to support the LLI during TiO_2_ sol–gel processing. These platforms helped during formed material post-characterization. We found that interfacially deposited TiO_2_ structures range from porous film obtained for low (<0.10%) and films decorated with NPs at high (>0.50%) TBOT % concentrations. The formation of TiO_2_ was further confirmed with EDX and infrared spectroscopy. In the future, we plan to investigate the possibility of further controlling the TiO_2_ sol–gel processing at the eLLI.

## Data Availability

Raw data can be found through the link: https://zenodo.org/record/6355563#.YjBZGnrMK3B (accessed on 10 February 2022).
